# Maternal immune activation generates anxiety in offspring: A translational meta-analysis

**DOI:** 10.1192/bjo.2021.714

**Published:** 2021-06-18

**Authors:** Ursula Matos, Laiana Azevedo Quagliato, Antonio Egidio Nardi

**Affiliations:** 1 Institute of Psychiatry, Federal University of Rio de Janeiro; 2 Full Professor of Psychiatry, Federal University of Rio de Janeiro

## Abstract

**Aims:**

Maternal immune activation (MIA) during pregnancy is recognized as an etiological risk factor for various psychiatric disorders, such as schizophrenia, major depressive disorder, and autism. Prenatal immune challenge may serve as a “disease primer” into an altered trajectory of fetal brain development that, in combination with other genetic and environmental factors, may ultimately result in the emergence of different psychiatric conditions. However, the association between MIA and the offspring's chances of developing anxiety disorders is less clear. To examine the effect of MIA on offspring anxiety, a systematic review and meta-analysis of the preclinical literature was conducted.

**Method:**

A systematic search of the PubMed, Web of Science, PsycINFO, and Cochrane Library electronic databases was performed using the PRISMA and WHO methodologies for systematic reviews. Studies that investigated if MIA during rodent's pregnancy could cause anxiety symptoms in offspring were included.

**Result:**

Overall, the meta-analysis showed that MIA induced anxiety behavior in offspring. The studies provide strong evidence that prenatal immune activation impacts specific molecular targets, synapse formation and function, and a disbalance in neurotransmission that could be related to the generation of offspring anxiety. Future research should further explore the role of MIA in anxiety endophenotypes.

**Conclusion:**

According to this meta-analysis, MIA plays an important role in the pathophysiological mechanisms of anxiety disorders and provides a promising therapeutic target.

